# PolyA-GLM: A comprehensive framework for *De novo* polyadenylation site prediction using genome language models

**DOI:** 10.1016/j.csbj.2025.12.011

**Published:** 2025-12-17

**Authors:** Sourav Saha, Naima Ahmed Fahmi, Jeongsik Yong, Wei Zhang

**Affiliations:** a Department of Computer Science, University of Central Florida, Orlando, FL, USA; b Department of Biochemistry, Molecular Biology and Biophysics, University of Minnesota Twin Cities, Minneapolis, MN, USA

**Keywords:** *De novo* polyadenylation site prediction, Genome language models, Transformer models, Few-shot learning, Post-transcriptional regulation, Cross-species testing

## Abstract

Polyadenylation sites (poly(A) sites) play a key role in the post-transcriptional regulation of gene expression. Accurate prediction of poly(A) sites is essential for identifying RNA processing defects associated with cancer and developmental disorders. Traditional approaches based on sequence motifs and experimental validation often struggle to generalize across different cell types and species. To address this limitation, we investigate the use of genome language models (GLMs) for poly(A) site prediction, leveraging their ability to capture long-range dependencies within genomic sequences. Specifically, we evaluate three state-of-the-art GLMs, DNABERT-2, Nucleotide Transformer, and HyenaDNA, using both few-shot classification and fine-tuning strategies. These models effectively recognize canonical polyadenylation signals (PASs) (i.e., AATAAA or other variants) and their spatial relationship (10-30 bp) to cleavage sites, with HyenaDNA achieving an AUC of 0.751 in the few-shot setting and improved performance after fine-tuning. We further validate model interpretability through systematic signal perturbation experiments, confirming their capacity to detect canonical PASs. Additionally, we propose a token-level classification approach for precise position-wise poly(A) site identification across extended gene regions. Finally, we present PolyA-GLM, an end-to-end pipeline for discovering novel poly(A) sites, highlighting the potential of GLMs to reveal regulatory elements overlooked by conventional methods. Overall, this work demonstrates the promise of GLMs in advancing our understanding of RNA processing and regulatory element discovery.

## Graphical Abstract

**Figure:**
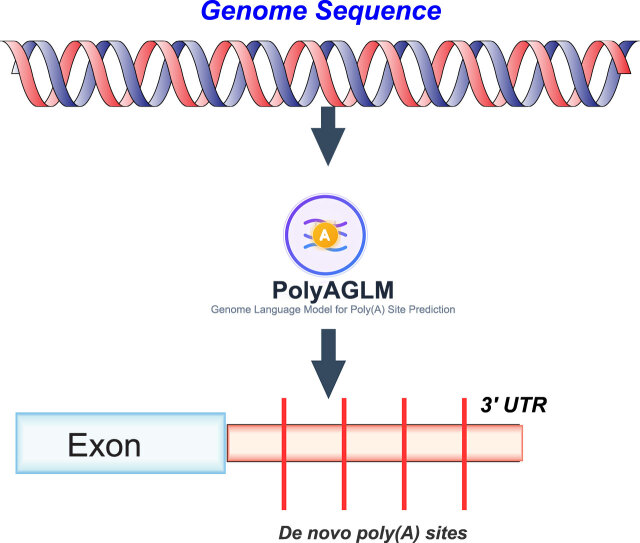

## 1 Introduction

Polyadenylation is a key mechanism in regulating gene expression at the post-transcriptional level in eukaryotes. During this process, the 3’-end of a messenger RNA (mRNA) transcript is cleaved and a polyadenylation (poly(A)) tail is added. This modification stabilizes the mRNA and regulates its export, translation, and eventual degradation [1], [2]. Errors in poly(A) site selection affect mRNA stability and alter protein expression levels, linking them to various developmental disorders and diseases, including cancer [3]. Aberrant intronic polyadenylation events have been observed in various cancers and can produce altered protein products that serve as tumor neoantigens [4]. As such, accurate detection and characterization of poly(A) sites are vital for understanding the molecular basis of gene regulation and disease [5], [6].

Traditional approaches for poly(A) site prediction have primarily relied on identifying conserved sequence motifs and experimental validation. Early computational methods focused on canonical PASs, such as the AATAAA hexamer and its known variants [7], [8], [9]. These approaches typically utilize position weight matrices (PWMs) and other statistical models to capture the distribution of cis-regulatory elements involved in polyadenylation. In parallel, several transcriptome-based computational tools were developed to directly detect and quantify alternative polyadenylation sites from RNA-seq data, though they remained constrained by the inherent limitations of short-read sequencing and rule-based heuristics [10], [11]. Subsequent advances incorporated machine learning techniques, such as support vector machines (SVMs) and random forests, which integrated additional features, including RNA secondary structure, nucleotide composition, and positional information [12], [13]. However, these methods remain limited in their ability to generalize across diverse cell types and species. Their reliance on handcrafted features and prior biological knowledge constrains their flexibility and sensitivity, particularly when applied to non-canonical PASs or underrepresented genomic contexts. Moreover, the performance of these models is often hindered by insufficient training data, leading to high false negative rates.

Accurately detecting poly(A) sites remains challenging due to the presence of long-range dependencies between the cleavage site and upstream PAS motifs, which are typically located 10–30 base pairs upstream of the poly(A) site [7], [8], [14]. To capture these long-range relationships, genome language models (GLMs) based on transformer and similar architectures are particularly well-suited. Several GLMs pre-trained on genomic sequences, including DNABERT [15], DNABERT-2 [16], Nucleotide Transformer (NT) [17], and HyenaDNA [18], have shown strong performance across a wide range of genomic tasks. These models, trained in an unsupervised manner on large-scale genomic data, are capable of learning complex nucleotide patterns, sequence motifs, and structural features of DNA. When fine-tuned on specific downstream tasks, they have demonstrated competitive performance in applications such as promoter classification [15], enhancer prediction [17], transcription factor binding site detection [15], splice site prediction [16], chromatin accessibility prediction [18], variant effect prediction [17], and lncRNA identification [19]. Recent benchmarking studies have shown that while GLM embeddings can approach the performance of task-specific expert methods on certain tasks, they may capture limited information about some long-range genomic features [20].

In this study, we explore the utility of GLMs for poly(A) site prediction. We design two experiments: one at the sequence level and another at the token level, to assess the models’ ability to identify poly(A) sites and their associated PASs. We evaluate the performance of GLMs using both few-shot classification and full fine-tuning approaches. Furthermore, we present PolyA-GLM, an end-to-end pipeline capable of identifying potential *de novo* poly(A) sites directly from genomic sequences. This work provides a comprehensive framework for poly(A) site prediction using GLMs and highlights their potential to uncover novel regulatory elements in the human genome.

## 2 Methods

Traditional approaches for poly(A) site prediction have primarily relied on identifying sequence motifs and incorporating experimental data [7], [21], [22]. However, recent advances in deep learning have introduced genome language models (GLMs) as powerful alternatives for sequence-based prediction tasks [23]. These models, typically built on transformer or other similar architectures, are pretrained to learn complex patterns and dependencies within DNA sequences [18], [24]. Several state-of-the-art GLMs have demonstrated strong performance in genomic tasks:

• DNABERT-2 [16] builds upon the original DNABERT framework by integrating byte pair embeddings and applying masked language model (MLM) pretraining on large-scale genomic datasets, resulting in improved performance across various downstream tasks.
• Nucleotide Transformer [17] employs a transformer-based architecture tailored for genomic data, with multiple variants pretrained on both human and multi-species genomes. It uses overlapping k-mer tokenization [25], representing sequences as overlapping 3-mers to capture local sequence patterns.
• HyenaDNA [18] adopts a novel long-range sequence modeling approach using state space models with the Hyena operator, a subquadratic alternative to attention mechanisms. This architecture helps with the efficient processing of very long genomic sequences, overcoming the computational bottlenecks of standard transformers.

**Fig. 1:**
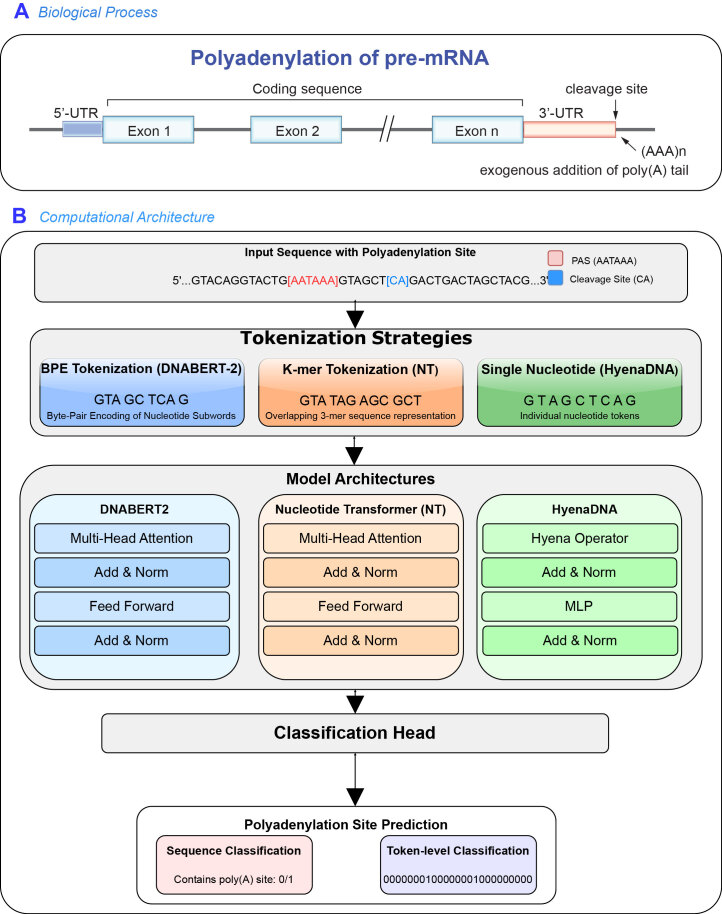
Framework for poly(A) site prediction in DNA sequences. (A) Polyadenylation involves recognition of the polyadenylation signal (PAS, typically AATAAA) and cleavage site in the 3’-UTR, followed by addition of a poly(A) tail. (B) The PolyA-GLM prediction pipeline processes input sequences containing PAS motifs and cleavage sites through three tokenization strategies: byte-pair encoding (DNABERT-2), overlapping k-mer representation (Nucleotide Transformer), and single-nucleotide tokens (HyenaDNA). Tokenized sequences are processed by transformer-based (DNABERT-2, NT) or convolution-based (HyenaDNA) architectures, with outputs fed to a classification head for either binary sequence-level prediction (contains poly(A) site: 0/1) or token-level prediction (only HyenaDNA) of exact site locations.

**Fig. 2:**
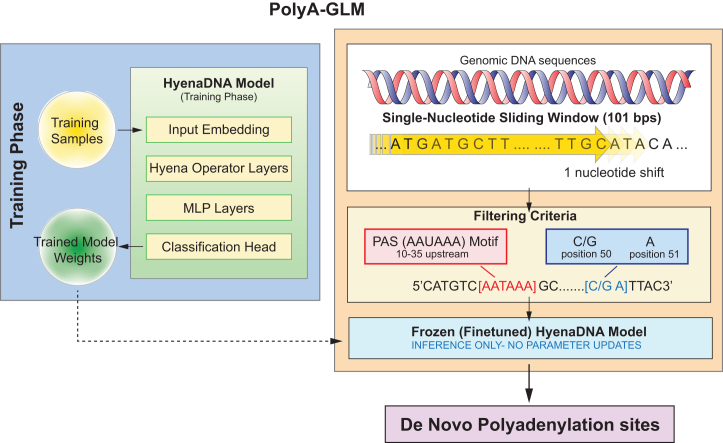
Framework for predicting *de novo* poly(A) sites using the HyenaDNA model. The approach consists of two phases. In the training phase, the model is trained on known polyadenylation signals (PASs) with all parameters updated. In the inference phase, finetuned model weights are frozen to ensure stable and consistent predictions. A single-nucleotide sliding window is applied across the genome, combined with specific filtering criteria, to scan for candidate *de novo* poly(A) sites. This framework leverages HyenaDNA’s long-range modeling capability and computational efficiency for genome-wide prediction.

Unlike traditional models that depend on manually engineered features, GLMs learn sequence-function relationships directly from raw DNA sequences through pre-training and fine-tuning, allowing them to model long-range genomic dependencies effectively. In this study, we introduce PolyA-GLM, a pipeline that leverages GLMs for poly(A) site prediction at both the sequence and token levels (Fig. 1), and further explore one of the GLMs’ (HyenaDNA’s) capabilities to identify *de novo* poly(A) sites (Fig. 2), applying its efficient computing performance.

### 2.1 An end-to-end framework for poly(A) site classification with genome language models

We created two balanced datasets, Gene-Gene and Gene-Intergene, to train and evaluate poly(A) site prediction models. Positive sites were obtained from the GENCODE v38 PolyA feature annotations provided by the UCSC Genome Browser [26].^1^ For each site, we extracted a 101 bp window, positioning the cleavage site at the 51st nucleotide and explicitly fixing that base to adenine (A). The same “51st-base-is-A” rule was applied to all negative examples to eliminate trivial single-base cues and to provide convolutional or attention-based models with a consistent coordinate system, ensuring that upstream PAS hexamers and downstream T/GT-rich elements always appear at the same relative positions [1], [27], [28].

Negative samples were collected under two conditions. In the **Gene-Gene** dataset, negatives were sampled from genic regions (within annotated genes) of each chromosome, ensuring these regions do not overlap with any GENCODE v38 polyA feature annotations from UCSC Genome Browser. Each negative sequence was required to (i) contain an adenine (A) at position 51 and (ii) include either ATTAAA or AATAAA, the two most prevalent PAS hexamers, within the 10–35 bp upstream region. This approach creates challenging negative examples that contain canonical PAS motifs in gene contexts but lack functional cleavage sites. The **Gene-Intergene** dataset used the same criteria for positives, but sourced its negatives from intergenic regions. After balancing, both datasets contained 50,182 positive and 50,182 negative sequences.

To assess model generalizability, we constructed mouse poly(A) site datasets following identical methodology using GENCODE annotations. Models fine-tuned on human data were evaluated directly on mouse sequences without additional training (see Supplementary Table 1).

To systematically assess model performance, we conducted five-fold cross-validation, partitioning each dataset into training (60 %), validation (20 %), and test (20 %) sets in each fold. Three specialized GLMs were applied to this task: DNABERT-2, Nucleotide Transformer, and HyenaDNA. After tokenization, the sequence embeddings were mean-pooled to generate a single vector representation for each input. We evaluated model performance using two approaches: (1) few-shot classification and (2) fine-tuning.

**Few-shot classification:** In the few-shot setting, we evaluated each pre-trained model’s ability to classify poly(A) sites without task-specific parameter updates. For each fold, we randomly selected two positive examples (poly(A) site containing sequences) and two negative examples (non-poly(A) sequences) from the training set to construct class prototypes. Each prototype vector was formed by averaging the mean-pooled embeddings from the selected examples in its class. These vectors are labeled as $p_{poly(A)}$ and $p_{non-poly(A)}$ respectively. For a query embedding $q_{i}$, we computed similarity scores to the two prototypes as follows:

(1) $$\begin{array}{l} {\text{sim}}_{(i)\text{poly(A)}}=1-\text{cosine}\left(q_{i},p_{\text{poly(A)}}\right),\\ {\text{sim}}_{(i)\text{non-poly(A)}}=1-\text{cosine}\left(q_{i},p_{\text{non-poly(A)}}\right) \end{array}$$

We then transformed these similarity values into a pseudo-probability for the poly(A) class using a softmax function:

(2) $$P_{i}=\frac{\exp({\text{sim}}_{(i)\text{poly(A)}})}{\exp({\text{sim}}_{(i)\text{poly(A)}})+\exp({\text{sim}}_{(i)\text{non-poly(A)}})}$$

Any query for which P_*i*_ exceeded 0.5 was labeled such that the sequence contains a poly(A) site (label=1), while the remainder were labeled such that the sequence doesn’t contain a poly(A) site (label=0). To reduce prototype selection bias (i.e., to exclude the dependence on extreme examples), this process was repeated 10 times per fold using different randomly selected prototype pairs, and the average performance was reported. To provide a comprehensive evaluation, we computed accuracy, precision, recall, F1-score, and the area under the ROC curve (AUC).

**Fine-tuning approach:** We fine-tuned each model for three epochs, with validation checks every 200 training steps. Early stopping was used to prevent overfitting, with a patience of five validation checks (i.e., training stopped if validation performance did not improve over five consecutive checks). A warmup-decay scheduler managed the learning rate, initialized at $10^{-6}$. Therefore, the total learning rate $\eta_{\text{t}}$ is defined as,

(3) $$\eta_{t}=\left\{ \begin{array}{ll} \eta_{\text{base}}\cdot \frac{t}{T_{\text{warmup}}} & \text{if }t<T_{\text{warmup}}\\ \eta_{\text{base}}\cdot \frac{1}{2}\left(1+\cos\left(\frac{\pi (t-T_{\text{warmup}})}{T_{\text{total}}-T_{\text{warmup}}}\right)\right) & \text{if }t\geq T_{\text{warmup}} \end{array}\right.$$

where $\eta_{\text{base}}=10^{-6}$ is the base learning rate, $T_{\text{warmup}}$ represents the number of warmup steps (set to 10 % of total training steps), and $T_{\text{total}}$ is the total number of training steps. During the warmup phase, the learning rate increases linearly from 0 to $\eta_{\text{base}}$. After warmup, it follows a cosine annealing schedule, gradually decreasing to 0 by the end of training.

For our experiments with validation checks every 200 training steps, we set:

(4) $$T_{\text{warmup}}=0.1\times N_{\text{epochs}}\times \left\lceil \frac{N_{\text{train}}}{B}\right\rceil$$

where $N_{\text{epochs}}=3$, $N_{\text{train}}$ is the number of training samples, and B=32 is the batch size.

All models were trained using the Binary Cross Entropy (BCE) loss:

(5) $$\text{BCE}=-\left(\frac{1}{N}\right)\sum_{i=1}^{N}\left[y_{i}\log(P_{i})+(1-y_{i})\log\left(1-P_{i}\right)\right]$$

where N is the number of samples in a batch, $y_{i}$ is the ground-truth label for the $i^{\text{th}}$ sample, and $P_{i}$ is the predicted probability. By adopting this framework, we aimed to develop a systematic and robust approach for accurately identifying poly(A) cleavage sites.

### 2.2 Genome-wide discovery of *de novo* poly(A) sites using fine-tuned genome language models

To identify potential poly(A) sites beyond annotated genomic coordinates, we applied a two-stage pipeline combining biological filtering with machine learning predictions to detect candidate *de novo* sites (Fig. 2).

**Stage 1 - Biological Filtering (Rule-based):** We applied a sliding window approach across the entire genome to detect candidate *de novo* sites. Each window spanned 101 bp, and a segment was considered a valid candidate only if it met the following three conditions: (1) an adenine (A) was present at the 51st position, (2) the nucleotide immediately preceding that adenine was either cytosine (C) or guanine (G), and (3) one of 18 PAS variants, derived from the two most common motifs AATAAA and ATTAAA [7], [29], was located between 10 and 35 bases upstream of the 51st position. These criteria were designed based on well-established biological observations. The requirement for an A at position 51 reflects the canonical cleavage preference, as pre-mRNA is typically cleaved immediately downstream of an adenosine residue [1], [30]. The second condition, requiring a C or G before the cleavage site, is supported by genomic analyses showing enrichment of CA and GA dinucleotides at authentic cleavage sites, which facilitate optimal positioning of the cleavage and polyadenylation specificity factor (CPSF) complex [31], [32]. The inclusion of 18 PAS variants, rather than limiting to the canonical AATAAA and ATTAAA, accounts for the biological diversity of functional PASs. Prior studies have shown that these variants collectively regulate approximately 30 % of polyadenylation events across different tissues [7], [33]. The included motifs were: AATAAA, ATTAAA, AGTAAA, CATAAA, TATAAA, GATAAA, ACTAAA, AATACA, AATATA, AAGAAA, AATAGA, AATGAA, TTTAAA, AAAATA, TATATA, AGATAA, ATTACA, and AGAATA. This broader motif set was chosen to capture a wider range of functional cleavage sites beyond the most prevalent signals. This filtering stage reduces the search space from ∼3 billion nucleotides in the human genome to a manageable set of biologically plausible candidates, dramatically reducing computational burden and false positives from random genomic scanning.

**Stage 2 - Model Prediction (Learning-based):** Each candidate sequence that satisfied the above biological criteria was then passed through our fine-tuned HyenaDNA model (see Fig. 1 sequence classification), which produced a probability score indicating whether the sequence resembled a true poly(A) site. Critically, this model evaluation step goes beyond simple motif presence. During training, the model learned that **PAS presence is necessary but not sufficient** for functionality; our negative training samples also contained canonical PAS motifs (AATAAA/ATTAAA) within the appropriate upstream region but lacked functional cleavage sites (see Section 2.1). Therefore, the model learned to evaluate additional contextual features that distinguish functional from non-functional sites, including: proper spacing relationships between regulatory elements, sequence composition in flanking regions, and other subtle patterns in the genomic context.

Recognizing that poly(A) sites can occur on both DNA strands, we repeated the same procedure on the reverse complement of each window to capture candidate sites on the negative strand. By systematically scanning the genome in this manner, we identified candidate *de novo* poly(A) sites that extend beyond current annotations. The model’s contribution lies in identifying which of the many PAS-containing genomic locations are likely to be functional poly(A) sites worthy of experimental validation.

### 2.3 Fine-grained poly(A) site identification through token-level modeling

We aimed to utilize HyenaDNA’s single-nucleotide tokenization to evaluate the positional prediction capabilities of GLMs. Using a dataset consisting of genomic sequences under 32,000 nucleotides in length (to control memory usage and computational overhead), we divided the data into standard training, validation, and test sets to ensure reproducibility.

In this setting, individual nucleotides (A, C, G, T) are treated as tokens within the language model framework (see Fig. 1), and each token is associated with a specific classification label. We adopted the HyenaDNA architecture, designed for efficient long-range sequence modeling, whose tokenization strategy enables position-level prediction at single-nucleotide resolution.

For model architecture, we added a dropout layer and a linear token-classification head on top of the HyenaDNA backbone, optimized for analyzing long genomic contexts. Training was performed using a weighted cross-entropy loss function that penalizes false negatives more heavily to address the imbalance between positive and negative labels.

This design leverages HyenaDNA’s ability to model long-range dependencies while improving sensitivity to the minority class. As a result, the model can deliver accurate, high-resolution predictions across extended genomic windows, enabling precise detection and characterization of poly(A) sites at the nucleotide level.

Performance evaluation included standard classification metrics (accuracy, precision, recall, and F1-score), as well as threshold-independent measures such as AUC. To account for the class imbalance, we also adjusted classification thresholds to optimize detection sensitivity while maintaining overall performance.

### 2.4 Validating biological feature learning through interpretability analysis

To assess whether the models learned biologically relevant features, we conducted two complementary interpretability experiments using fine-tuned models.

**Perturbation Analysis:** We systematically disrupted canonical PAS motifs (AATAAA and ATTAAA) in positive test samples to evaluate model reliance on these signals. For each perturbation level p∈{10%,20%,…,100%}, we applied the following procedure:

(6) $$S_{\text{perturbed}}=\left\{ \begin{array}{ll} S_{\text{original}} & \text{if }r>p\\ \text{Replace}(S_{\text{original}},M,R) & \text{if }r\leq p \end{array}\right.$$

where $S_{\text{original}}$ is the original sequence, M represents the PAS motif (AATAAA or ATTAAA), R is a random nucleotide sequence of equal length, and r is a uniform random variable determining whether each individual PAS motif is perturbed. Each perturbed sequence($P_{i}$) was then passed through the fine-tuned HyenaDNA model to obtain predicted probabilities $P_{i}\in [0,1]$. We evaluated model performance using two metrics: (1) the distribution of predicted probabilities across all perturbed samples, and (2) the misprediction count at each perturbation level:

(7) $$N_{\text{mispred}}(p)=\sum_{i=1}^{N}(P_{i}<0.5)$$

where N is the total number of test samples.

**Attention Analysis:** Using the fine-tuned Nucleotide Transformer model, we extracted attention weights from the final transformer layer for all positive test samples. For each sequence, we identified the single token (i.e., each token spans 6 bp due to taking 6-mer tokenization) with the highest attention score. We then aggregated these top-attended 6-mers across all samples to compute their frequencies. The most frequently attended 6-mers were compared against known canonical PAS sequences (AATAAA, ATTAAA) and other regulatory elements to assess whether the model learned biologically relevant features.

## 3 Results

### 3.1 Model performance: few-shot capabilities and fine-tuning improvements

We evaluated three pretrained GLMs (DNABERT-2, Nucleotide Transformer, and HyenaDNA) across two training approaches to assess their ability to identify poly(A) sites. These models were pre-trained on large genomic datasets and tested using two complementary approaches: few-shot classification (using the models’ inherent knowledge without additional training) and fine-tuning (adapting the models specifically for poly(A) site prediction).

We evaluated the models using two balanced datasets (Gene-Gene and Gene-Intergene) with 50,182 positive and negative sequences each, where negative sequences contained canonical motifs but lacked functional cleavage sites (see Methods). The comprehensive performance comparison across these conditions is shown in Table 1.

**Table 1:** Performance comparison of genome language models on poly(A) site detection using 5-fold cross-validation. The table presents the results of detecting poly(A)-site-containing sequences on two different datasets. In the first dataset, the negative classes come from gene regions (G-G; Gene-Gene), while in the second dataset, they come from intergenic regions (IG-G; Gene-Intergene) (see Methods for details on dataset creation). We report the average results from five-fold cross-validation here.

		Acc.		Precision		Recall		F1 Score		AUC	
Method	Model	G-G	IG-G	G-G	IG-G	G-G	IG-G	G-G	IG-G	G-G	IG-G
Few-shot	DNABERT-2	0.4994	0.4787	0.4997	0.484	0.8885	0.6651	0.6396	0.5606	0.4876	0.4650
	Nucleotide Transformer (500 M)	0.6043	0.5967	0.6626	0.6472	0.4252	0.4252	0.5180	0.5132	0.6801	0.6723
	HyenaDNA	0.6769	0.6988	0.6896	0.7235	0.6434	0.7235	0.6657	0.6811	0.7510	0.7541
Fine-tune	DNABERT-2	0.7638	0.7694	0.7661	0.7709	0.7638	0.7694	0.7632	0.7691	0.7638	0.7694
	Nucleotide Transformer (100 M)	0.7862	**0.7855**	0.7864	**0.7861**	0.7862	**0.7855**	0.7862	**0.7854**	0.7862	**0.7855**
	Nucleotide Transformer (500 M)	**0.7967**	0.7751	**0.7978**	0.7777	**0.7967**	0.7751	**0.7965**	0.7745	**0.7967**	0.7751
	HyenaDNA	0.7606	0.7692	0.7618	0.7713	0.7606	0.7692	0.7603	0.7687	0.7606	0.7692

**Strong few-shot performance without task-specific training:** Even without task-specific training, two of the three models demonstrated substantial ability to distinguish functional poly(A) sites from similar-looking sequences. As detailed in Table 1, HyenaDNA achieved the strongest performance with an AUC of 0.751 on both datasets Gene-Gene (where negative sequences come from gene regions) and Gene-Intergene (where negatives come from intergenic regions). Nucleotide Transformer also performed well with AUCs of 0.6801 and 0.6723, respectively. DNABERT-2 performed poorly with AUCs around 0.49, essentially at random chance levels. Both HyenaDNA and Nucleotide Transformer showed significant performance without task-specific training. This suggests that models learned biological significance (possibly ATTAAA and AATAAA PAS motifs) and adenine cleavage sites, even without task-specific training. HyenaDNA’s performance probably stemmed from its ability to capture long-range dependencies, while Nucleotide Transformer’s success resulted from its larger model scale, both of which helped to achieve superior performance [17], [18].

**Task-specific finetuning improves performance:** Task-specific fine-tuning improved performance for all models, as shown in Table 1. The largest Nucleotide Transformer variant (500 M parameters) achieved the highest performance: AUCs of 0.80 (Gene-Gene) and 0.78 (Gene-Intergene). Other models showed consistent improvements, reaching AUCs between 0.76–0.79 across both datasets. The substantial gains from fine-tuning demonstrate that while pre-trained models capture relevant biological patterns, focused training on poly(A) sites enables them to learn more precise recognition criteria. Notably, even modest amounts of labeled data (approximately 60,000 training sequences per fold) were sufficient to achieve these improvements, suggesting that the models’ pre-existing biological knowledge provides an effective foundation for specialized tasks.

**Cross-species generalization confirms biological feature learning:** To validate that our models learned evolutionarily conserved polyadenylation features rather than human-specific patterns, we evaluated the human-trained models directly on mouse poly(A) sites without any additional training (Supplementary Table 1). All models demonstrated robust cross-species performance, with Nucleotide Transformer (500 M) achieving AUC scores of 0.7547 (Gene-Intergene) and 0.7437 (Gene-Gene), while DNABERT-2 and HyenaDNA achieved AUCs ranging from 0.66 to 0.71. The high recall rates (74–82 %) across all models indicate strong sensitivity to canonical polyadenylation signals conserved between species. Notably, the cross-species performance approached our few-shot learning results on human data, suggesting that the models successfully captured fundamental polyadenylation mechanisms.

### 3.2 Model interpretability: perturbation and attention analysis validate biological learning

We conducted two experiments, signal perturbation (HyenaDNA) and attention analysis (NT-500 M), to assess whether the models captured the underlying biological relevance. Both experiments indicated that the models learned the dependence on the underlying PAS for poly(A) site prediction.

**Fig. 3:**
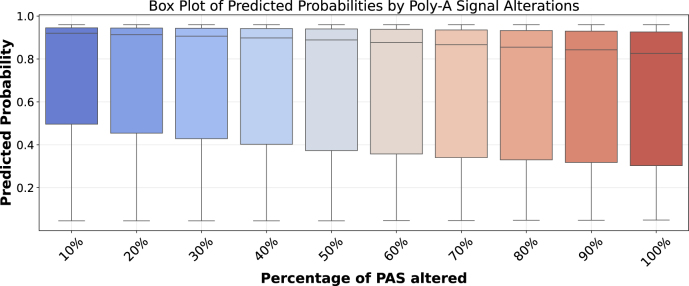
Box plot showing the distribution of predicted probabilities on positive class across different levels of PAS alteration. The model exhibits progressively declining confidence factor (predicted probability) as more PASs are disrupted, illustrating the model’s reliance on canonical PASs for accurate classification.

**Fig. 4:**
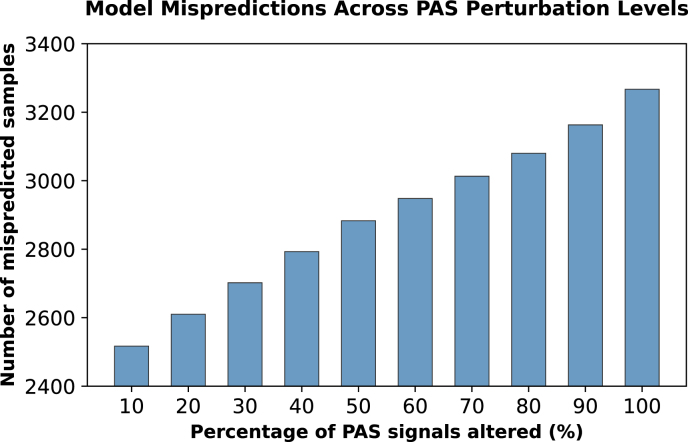
Number of mispredicted samples across increasing error rates. A broken y-axis is used to emphasize variations in misprediction counts, which range from 2517 to 3267 out of a total of 10,038 samples.

**Signal perturbation reveals dependency on canonical motifs:** We systematically replaced canonical AATAAA and ATTAAA motifs with random sequences at varying levels to test model dependence on these critical signals, illustrated by Fig. 3, Fig. 4. As more PASs were disrupted, model performance declined correspondingly, with predicted probability scores showing a progressive decrease (Fig. 3). These probability scores reflect the likelihood assigned by the model to each sequence being a functional poly(A) site, where scores approaching 1.0 indicate strong prediction of polyadenylation activity and scores near 0.0 suggest that the model is confident that the sequence doesn’t have any functional poly(A) site. The corresponding increase in mispredictions, from approximately 2500 to over 3200 samples as perturbation levels increased, confirms the models’ genuine reliance on these canonical elements (Fig. 4). This pattern shows the models genuinely rely on these canonical PAS rather than picking up on unrelated sequence patterns. The progressive decline reflects biological signal disruption rather than out-of-distribution (OOD) confusion. Our negative samples contain PAS motifs but lack complete functional architecture, demonstrating the model learned that PAS is necessary but not sufficient for functional sites. The smooth degradation across perturbation levels confirms systematic response to biological signal loss rather than erratic OOD behavior.

**Fig. 5:**
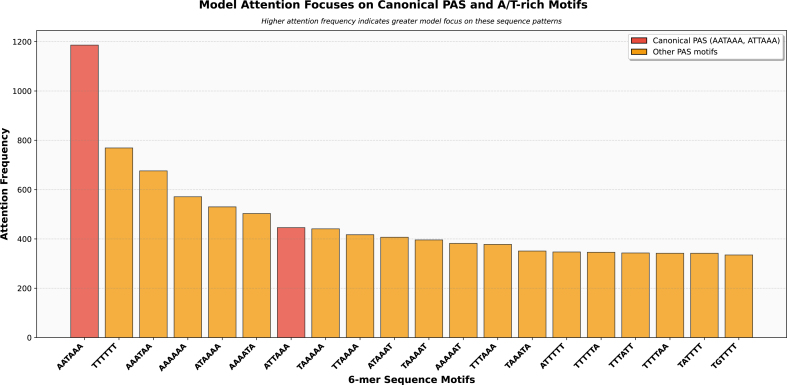
Analysis of most attended 6-mer tokens in positive poly(A) site sequences. Bar chart showing the frequency distribution of the top 20 most attended 6-mer tokens by the genome language model when processing sequences containing poly(A) sites. The y-axis represents the frequency of each 6-mer token appearing among the top 50 most attended positions across all positive samples. It is notable that the top attended motif are either canonical PASs (AATAAA, ATTAAA) or other known A/T enriched variants, demonstrating the NT-500 M model’s ability to focus attention on biologically relevant sequence motifs without explicit supervision.

**Attention analysis confirms focus on relevant motifs:** Analysis of model attention patterns in Fig. 5 revealed that the models consistently focus on canonical PASs and their characteristic surrounding sequences. When examining the most frequently attended 6-mer sequences in positive samples, AATAAA emerged as the most attended motif, followed by various adenine-rich and thymine-rich patterns characteristic of 3’ UTR processing regions.

This attention pattern aligns precisely with known poly-adenylation biology: the canonical AATAAA or other similar signals typically appear 10–30 nucleotides upstream of cleavage sites, surrounded by adenine-rich upstream elements that facilitate proper 3’-end processing. The models’ ability to identify and focus on these elements without explicit instruction demonstrates their capacity to learn meaningful biological relationships from sequence data alone.

In this study, we limited our attention analysis to sequences with experimentally supported poly(A) sites to examine where the model focuses when predicting functional sites. A comparison of attention patterns on PAS motifs in negative, PAS-containing sequences remains an important direction for future work, which is consistent with recent work showing that attention over the same motif can vary strongly with sequence context in genomic transformer models [34].

### 3.3 Token-level classification achieves high precision despite class imbalance

We applied token-level classification to identify individual nucleotides corresponding to poly(A) sites across genomic sequences totaling 50,298,251 tokens in the test set. Among these, 11,199 positions corresponded to true poly(A) sites (0.022 % of all positions), reflecting their extreme rarity in genomic DNA.

The model correctly identified 1802 poly(A) sites, yielding a recall of 16.0 %. Of the positions predicted as potential sites, 1802 were true positives and 3260 were false positives, corresponding to 35.6 % precision. For the abundant negative class, the model correctly classified 50,283,792 positions as non-poly(A) sites, achieving 99.99 % specificity.

While the 16.0 % recall appears low, this performance must be interpreted within the context of genomic sequence analysis. Poly(A) sites represent roughly 1 in 4500 nucleotides, making them extremely difficult to detect. The 35.6 % precision represents substantial improvement over random selection, which would achieve only 0.022 % precision. This translates to approximately 1600-fold enrichment, making the approach useful for prioritizing regions for experimental validation.

The overall accuracy of 99.975 % primarily reflects the correct classification of the abundant negative class. More importantly, rather than making simple yes/no predictions, the model assigns probability scores to each position. When we sort all genomic positions by these probability scores from highest to lowest, the true poly(A) sites cluster near the top of this ordered list. The AUC of 0.8711 measures how well the model can order positions this way - a perfect score of 1.0 would mean all true sites appear at the very top when sorted by probability. This scoring system allows for examining the most promising candidates first, even if the model cannot perfectly classify every position.

### 3.4 Genome-wide prediction of novel poly(A) sites

We applied our two-stage pipeline to identify *de novo* poly(A) sites across the entire human genome beyond current annotations.

**Fig. 6:**
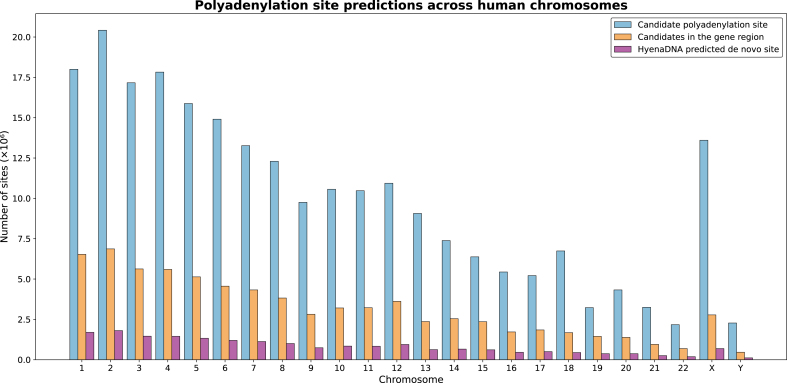
Chromosome-wise distribution of *de novo* polyadenylation site (poly(A) site) predictions. The figure shows three categories: candidate sequences meeting biological criteria (Blue), Candidates in the gene region (Orange), and predicted sites by HyenaDNA (Pink). (For interpretation of the references to colour in this figure legend, the reader is referred to the web version of this article.)

**Stage 1 - Biological Filtering:** Using strict sequence criteria (adenine at position 51, C/G immediately upstream, and one of 18 validated PAS variants 10–35 bp upstream), we identified candidate sequences distributed across all genomic regions (Fig. 6, blue bars). This filtering step reduced the search space from ∼3 billion nucleotides to biologically plausible candidates while maintaining sensitivity to diverse PAS motifs.

**Stage 2 - Model Evaluation:** Our fine-tuned HyenaDNA model evaluated each candidate, assigning probability scores to distinguish likely functional sites from non-functional PAS-containing sequences. Importantly, the model’s training on negative examples (which also contained canonical PAS motifs but lacked functional cleavage sites) enabled it to learn contextual features beyond simple motif presence. The model’s predicted poly(A) sites within genic regions (Fig. 6) are consistent with the expected distribution of functional poly(A) sites.

**Fig. 7:**
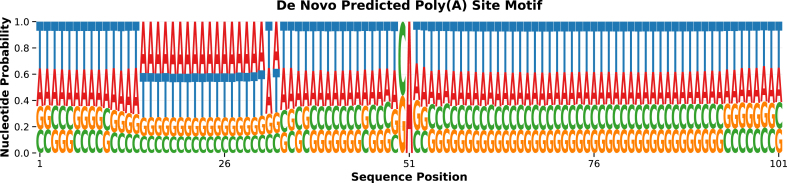
Motif logo generated from the *de novo* predicted poly(A) site sequences.

**Validation of predicted sites:** Analysis of the predicted sites confirmed they contain canonical polyadenylation features. Motif logos revealed PASs positioned upstream of predicted cleavage sites with proper spatial relationships (Fig. 7). This validation serves two critical purposes: (1) it confirms that high-scoring predictions maintain biologically expected sequence architecture rather than representing random selections from the candidate pool, and (2) it verifies that the model learned genuine regulatory patterns. The presence of these canonical features at proper positions indicates that the model successfully learned to evaluate the complete functional architecture of poly(A) sites, not just motif presence, but also appropriate spacing, sequence composition, and other contextual patterns that distinguish functional from non-functional sites.

**Interpretation of “*de novo*” predictions:** These predicted sites are considered “novel” or “*de novo*” because they are predicted by genome language models, not because they lack canonical sequence features. Indeed, the presence of expected PAS motifs and proper spatial organization validates rather than contradicts their potential functionality. The model’s contribution is prioritizing which of the many PAS-containing genomic loci warrant experimental validation as potential functional poly(A) sites. These predictions represent testable hypotheses that could be validated experimentally through 3’-end sequencing techniques (e.g., PAS-seq, PolyA-seq) to confirm their functional activity in specific tissues or conditions.

## 4 Discussions

Our results demonstrate that GLMs can identify poly(A) sites without explicit task-specific training. This suggests that PASs occur frequently enough in genomic DNA for models to capture them during general pre-training. Among the comparisons, HyenaDNA outperformed transformer-based models, likely due to its ability to process longer sequences more efficiently. While PAS and cleavage sites are typically separated by only 10–30 bp, the surrounding context may contain additional regulatory elements that influence polyadenylation. When we systematically replaced canonical PAS with random sequences, model performance dropped in a way that mirrors experimental work showing AATAAA and ATTAAA mutations reduce polyadenylation efficiency [30]. This indicates the models rely on actual biological signals rather than picking up irrelevant sequence patterns. Such perturbation experiments could help determine which sequence features matter for predictions in other genomic applications.

GLMs require substantially more computational resources than traditional sequence analysis methods. For poly(A) site prediction, simpler approaches may provide adequate performance at much lower costs. But once the model is trained, it can predict a new sequence at a faster rate at the inference level and doesn’t need to go through the whole process again. The trade-off between performance gains and computational requirements needs careful evaluation. The extreme class imbalance in genomic data presents ongoing challenges for token-level prediction, despite the demonstrated ranking capabilities of our approach. A more fine-grained approach to tackle the positional predictability of the poly(A) sites using a long sequence is a good challenge for future work.

To assess whether our models learned evolutionarily conserved features, we evaluated human-trained models on mouse poly(A) sites without any mouse-specific training (Supplementary Table 1). All models demonstrated robust cross-species generalization well above random chance, with Nucleotide Transformer (500 M) achieving AUC scores of 0.7547 (Gene-Intergene) and 0.7437 (Gene-Gene), while DNABERT-2 and HyenaDNA achieved AUCs of 0.66–0.71. Notably, all models maintained high recall rates (74 %–82 %), and the cross-species performance matched or approached our few-shot learning results on human data (HyenaDNA: 0.75 AUC), suggesting that fine-tuning on human sequences effectively captures core polyadenylation mechanisms conserved across mammalian evolution. The modest performance difference between within-species (AUC: 0.76–0.80) and cross-species evaluation likely reflects genuine biological variation in species-specific regulatory contexts rather than fundamental model limitations. These results validate that our models learn biologically meaningful, evolutionarily conserved features rather than species-specific artifacts, supporting their potential application to poly(A) site prediction in other organisms where training data may be limited.

The success of GLMs at learning polyadenylation patterns suggests they may be useful for other regulatory element prediction tasks. Their ability to capture sequence-function relationships without manual feature engineering could be valuable for discovering regulatory elements more broadly. However, generalizability across species and tissues needs validation. Most importantly, the biological functionality of predicted sites requires experimental confirmation. Looking forward, the catalog of predicted *de novo* poly(A) sites generated by PolyA-GLM can support downstream analyses of regulatory variation. These sites may be integrated with tissue-resolved or disease-specific RNA-seq datasets to identify tissue-enriched patterns of alternative polyadenylation. Such integration could also reveal regulatory changes associated with pathological 3’-UTR remodeling.

To assess the biological relevance of our *de novo* predictions, we compared our results against PolyASite 2.0 [28], a comprehensive database of experimentally validated poly(A) sites. We found that 437,291 (77 %) of our predictions overlap within ±20 nucleotides of PolyASite 2.0 annotations. This window is biologically meaningful, as poly(A) sites exhibit well-documented micro-heterogeneity with functional sites clustering within ±12 nucleotides and spanning up to 25 nucleotides due to imprecise cleavage by the 3’-end processing machinery [28]. In addition, we performed a similar analysis using our independent 3’-end sequencing (3’-end-seq) datasets [35], [36], [37] (see Supplementary Document)

The precision-recall characteristics of our approach reflect deliberate design choices for genome-wide discovery. With our best model achieving an AUC of 0.80, we prioritized sensitivity to capture potential novel sites while maintaining biological plausibility through strict sequence requirements. Predictions not overlapping with PolyASite 2.0 may represent tissue-specific or condition-dependent sites not captured in current databases, cryptic sites with lower usage frequency, or false positives requiring experimental validation. The enrichment of our predictions within genic regions versus intergenic regions (Fig. 6) provides orthogonal validation that our model captures biologically relevant patterns. This genome-wide discovery approach parallels strategies used for other regulatory elements, where computational methods generate candidate sets for targeted experimental validation.

**Limitations:** While our approach demonstrates promising results, several important limitations should be noted. First, our models analyze DNA sequences only and cannot capture RNA-level regulatory features such as RNA secondary structure, RNA-binding protein interactions, or co-transcriptional regulatory mechanisms that influence poly(A) site selection *in vivo*. Second, our *de novo* poly(A) site predictions, while containing expected sequence features (Fig. 7), require experimental validation through 3’-end-seq techniques to confirm functional activity. Third, our models were trained and evaluated exclusively on human genomic sequences; generalization to other species requires systematic validation. Finally, GLMs require substantially greater computational resources than traditional motif-based methods during the training phase, though inference is efficient once models are trained. Additionally, our token-level classifier operates across very long genomic windows, making training computationally expensive and particularly sensitive to extreme class imbalance; more advanced balancing or sampling strategies may further improve positional recall.

## 5 Conclusion

This research shows the experimental success of GLMs for poly(A) site prediction, revealing their ability to capture complex sequence patterns and long-range dependencies essential for accurate identification of these critical regulatory elements. The impressive performance of HyenaDNA in few-shot classification tasks, combined with robust cross-species generalization to mouse genomes, indicates that these models may inherently learn evolutionarily conserved and biologically relevant features during pre-training, even without explicit task-specific instruction. Our fine-tuning experiments further enhance these capabilities, indicating further scope for improvement through training with task-specific supervised data. The identification of potential novel poly(A) sites demonstrates the practical utility of these approaches for expanding our understanding of the transcriptome.

Future work should incorporate multi-modal data integration, including tissue-specific expression patterns. Exploring hybrid model architectures that combine the strengths of different GLM approaches could also yield advances in performance. Overall, this work establishes GLMs as powerful tools for understanding post-transcriptional regulation and highlights their potential for discovering previously uncharacterized functional elements in the human genome, with implications for both basic research and clinical applications in diseases associated with aberrant RNA processing.

## CRediT authorship contribution statement

**Sourav Saha:** Writing – original draft, Visualization, Validation, Software, Resources, Methodology, Investigation, Formal analysis, Data curation, Conceptualization. **Naima Ahmed Fahmi:** Writing – original draft, Resources, Conceptualization. **Jeongsik Yong:** Writing – original draft, Supervision, Conceptualization. **Wei Zhang:** Writing – original draft, Supervision, Methodology, Investigation, Funding acquisition, Conceptualization.

## Data Availability

The source code for the models and the constructed dataset is available at https://github.com/compbiolabucf/PolyA_GLM.

## Footnotes

1 https://www.gencodegenes.org/human/release_38.html
