# Supplementary material for: PolyA-GLM: A comprehensive framework for De novo polyadenylation site prediction using genome language models
Source: Comput Struct Biotechnol J. 2025 Dec 17;31:120–9. doi: 10.1016/j.csbj.2025.12.011 (PMC12799945; doi:10.1016/j.csbj.2025.12.011)
Supplement: Multimedia Component 1 [file mmc1.pdf]

Supplementary Documents

Cross Species Dataset Result

Table 1: Cross-species evaluation results: models trained on human datasets and evaluated on mouse datasets.

Dataset	Model	Accuracy	Precision	Recall	F1-score	AUC
Gene–Intergene	HyenaDNA	0.6291	0.5930	**0.8238**	0.6896	0.6580
	Nucleotide Transformer (500M)	**0.6917**	**0.6728**	0.7464	**0.7077**	**0.7547**
	DNABERT-2	0.6401	0.6108	0.7724	0.6822	0.7029
Gene–Gene	HyenaDNA	0.6513	**0.6270**	0.7470	0.6818	0.6938
	Nucleotide Transformer (500M)	**0.6809**	0.6600	0.7464	**0.7005**	**0.7437**
	DNABERT-2	0.6369	0.6056	**0.7850**	0.6837	0.7143

Few-Shot Prototype Result

Table 2: Prototype-based few-shot results for HyenaDNA on the Gene–Gene (G-G) dataset.

n_prototypes	Accuracy	Precision	Recall	F1	AUC
1	0.5991	0.6543	0.4203	0.5118	0.6662
2	0.6718	0.6836	0.6396	0.66089	0.7447
3	0.6012	0.6303	0.4895	0.5510	0.6660
4	0.6331	0.6235	0.6719	0.6469	0.6866
5	0.6774	0.6796	0.6714	0.6755	0.7359

Table 3: Prototype-based few-shot results for HyenaDNA on the Intergene–Gene (IG-G) dataset

n_prototypes	Accuracy	Precision	Recall	F1	AUC
1	0.6238	0.7086	0.4203	0.5277	0.7189
2	0.6990	0.7260	0.6396	0.6800	0.7840
3	0.6568	0.6865	0.5772	0.6271	0.7373
4	0.6833	0.6250	0.9165	0.7432	0.7868
5	0.6966	0.6900	0.7142	0.7019	0.7655

Table 4: Prototype-based few-shot results for the Nucleotide Transformer (NT) model on the Gene–Gene (G-G) dataset.

n_prototypes	Accuracy	Precision	Recall	F1	AUC
1	0.6087	0.6861	0.4004	0.5057	0.6892
2	0.6010	0.6560	0.4249	0.5157	0.6731
3	0.6239	0.6687	0.4910	0.5662	0.6988
4	0.6080	0.6310	0.5203	0.5703	0.6659
5	0.6251	0.6530	0.5338	0.5874	0.6846

Table 5: Prototype-based few-shot results for the Nucleotide Transformer (NT) model on the Intergene–Gene (IG-G) dataset.

n_prototypes	Accuracy	Precision	Recall	F1	AUC
1	0.6087	0.6861	0.4004	0.5058	0.6892
2	0.6010	0.6560	0.4249	0.5157	0.6731
3	0.6239	0.6687	0.4910	0.5662	0.6988
4	0.6080	0.6310	0.5203	0.5703	0.6659
5	0.6251	0.6530	0.5338	0.5874	0.6846

Table 6: Prototype-based few-shot results for DNABERT-2 on the Gene–Gene (G-G) dataset.

n_prototypes	Accuracy	Precision	Recall	F1	AUC
1	0.5638	0.6064	0.3637	0.4547	0.6062
2	0.6277	0.6338	0.6048	0.6190	0.6756
3	0.6314	0.6568	0.5502	0.5988	0.6863
4	0.6019	0.6012	0.6050	0.6031	0.6372
5	0.6281	0.6197	0.6633	0.6407	0.6761

Table 7: Prototype-based few-shot results for DNABERT-2 on the Intergene–Gene (IG-G) dataset.

n_prototypes	Accuracy	Precision	Recall	F1	AUC
1	0.5690	0.6170	0.3637	0.4576	0.6183
2	0.6408	0.6517	0.6048	0.6274	0.6947
3	0.6390	0.6121	0.7588	0.6776	0.6999
4	0.6480	0.6327	0.7060	0.6673	0.7020
5	0.6490	0.6486	0.6502	0.6493	0.7056

Validation Against PolyASite 2.0

We performed a comprehensive comparison of our de novo poly(A) site predictions with PolyASite 2.0 annotations across all human chromosomes. The ± 20 nucleotide (nt) window was chosen based on established literature documenting poly(A) site heterogeneity. According to the PolyASite2.0 paper, poly(A) sites within ± 12 nt are typically considered the same functional site, with clusters spanning up to 25 nt. Our analysis at two thresholds shows:

- 33.4% overlap within ±5 nt
- 76.9% overlap within ±20 nt

This progressive increase reflects the biological reality.

Validation Against 3'-end-seq Data

To further validate the biological relevance of our de novo predicted poly(A) sites, we quantified their overlap with experimentally derived 3'-end peaks from the 3'-end-seq datasets of MCF7 and BT549 from our previous studies [1, 2, 3]. Across the combined datasets, we identified 72,684 distinct 3'-end peaks. We then evaluated the proximity of PolyA-GLM predictions to these experimentally supported cleavage sites.

Using increasingly permissive matching windows, we observed the following overlap rates:

- ±5 nt window: 6,400 overlaps (8.81%)
- ±20 nt window: 14,243 overlaps (19.60%)
- ±50 nt window: 20,026 overlaps (27.55%)

These results demonstrate that a substantial fraction of our predicted poly(A) sites fall within a biologically meaningful distance of 3'-end peaks, with overlap increasing systematically as the window expands. The concordance at ±20 nt aligns with known micro-heterogeneity of polyadenylation cleavage, while the broader ±50 nt window captures additional experimentally supported but variable cleavage events. Collectively, these findings provide strong experimental support for the biological validity of the de novo sites identified by PolyA-GLM.

Accuracy & AUC drop With Perturbation

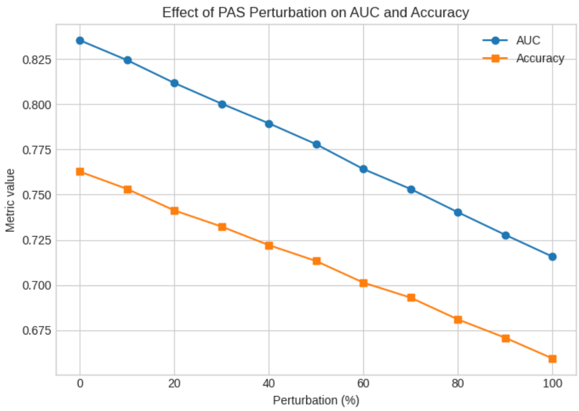

Figure 1: AUC & accuracy drop due to perturbation. Note that this AUC & accuracy experiments were done with one fold rather than the average of 5 folds mentioned in the paper.

References

[1] Jiao Sun, Jin-Young Kim, Semo Jun, Meeyeon Park, Ebbing de Jong, Jae-Woong Chang, Sze Cheng, Deliang Fan, Yue Chen, Timothy J Griffin, et al. Dichotomous intronic polyadenylation profiles reveal multifaceted gene functions in the pan-cancer transcriptome. *Experimental & Molecular Medicine*, 56(10):2145–2161, 2024.

[2] Sze Cheng, Naima Ahmed Fahmi, Meeyeon Park, Jiao Sun, Kaitlyn Thao, Hsin-Sung Yeh, Wei Zhang, and Jeongsik Yong. mTOR contributes to the proteome diversity through transcriptome-wide alternative splicing. *International journal of molecular sciences*, 23(20):12416, 2022.

[3] Naima Ahmed Fahmi, Sze Cheng, Jeovani Overstreet, Qianqian Song, Jeongsik Yong, and Wei Zhang. IPScan: Detecting novel intronic PolyAdenylation events with RNA-seq data. *PLOS Computational Biology*, 21(11):e1013668, 2025.
